# The Detergent-Soluble Cytoplasmic Pool of Survivin Suppresses Anoikis and Its Expression Is Associated with Metastatic Disease of Human Colon Cancer

**DOI:** 10.1371/journal.pone.0055710

**Published:** 2013-02-06

**Authors:** Masato Hori, Tomoharu Miki, Mayumi Okamoto, Futoshi Yazama, Hiroaki Konishi, Hiroshi Kaneko, Fumio Shimamoto, Takahide Ota, Achim Temme, Masaaki Tatsuka

**Affiliations:** 1 Department of Life Sciences, Faculty of Life and Environmental Sciences, Prefectural University of Hiroshima, Shoubara, Hiroshima, Japan; 2 Department of Health Sciences, Faculty of Human Culture and Science, Prefectural University of Hiroshima, Minami-ku, Hiroshima, Japan; 3 Department of Life Science, Medical Research Institute, Kanazawa Medical University, Uchinada, Ishikawa, Japan; 4 Department of Neurosurgery, University Hospital Carl Gustav Carus, Technical University Dresden, Dresden, Germany; IISER-TVM, India

## Abstract

Survivin is a component of the chromosomal passenger complex (CPC) that is essential for accurate chromosome segregation. Interfering with the function of Survivin in mitosis leads to chromosome segregation errors and defective cytokinesis. Survivin contains a Baculovirus IAP Repeat (BIR) and therefore was originally classified as inhibitor of apopotosis protein (IAP), yet its role in apoptosis after cellular stress remains largely unknown. We demonstrate here, that Survivin predominantly suppresses anoikis, a form of programmed cell death induced by loss of cellular adhesion to extracellular matrix. Interestingly, cells ectopically overexpressing EGFP-Survivin showed after loss of cell-matrix-interaction a decreased expression of IκB-α. Subsequent subcellular protein fractionation and immunoprecipitation experiments revealed that XIAP interacts with detergent-soluble Survivin which is known to cooperatively activate NF-κB signaling. Examination of the expression levels of detergent soluble Survivin in colorectal cancer cell lines and in colorectal cancerous tissues revealed that detergent soluble cytoplasmic Survivin levels correlated inversely with anoikis susceptibility in colorectal cancer. Therefore, the detergent soluble cytoplasmic Survivin might be a promising predictive biomarker for lymph node and distant metastases of colorectal cancer. We conclude that an anti-apoptotic function of detergent-soluble Survivin in interphase cells experiencing anoikis is mediated at least via XIAP/IκB-α/NF-κB signaling.

## Introduction

During cancer development, metastatic cells detach from neighboring tumor cells, acquire cell motility, invade and enter the lymph system or blood circulation, survive and form metastatic lesions. These steps involve many genes and pathways, and yet these biological processes are poorly understood despite many scientific approaches [1]. The identification of metastatic tumor-targeting molecules for diagnostic and therapeutic procedures is still required.

Survivin is a member of the chromosomal passenger protein complex (CPC) that is a key regulator of mitosis. Survivin and other CPC components, Aurora-B, inner centromere protein (INCENP), and Borealin (Dasra B) are essential for the CPC functions including kinetochore attachment error corrections and completion of cytokinesis [2]. Survivin contributes to the mitotic localization of the CPC and has been described to enhance Aurora-B kinase activity as shown in *Xenopus laevis* and *S. pombe* [3], [4]. In early mitosis, phosphorylation of Histone H3 at threonine 3 mediated by Haspin and subsequent binding of Survivin to this site is critical for assembling CPC on kinetochores [5], [6], [7].

Survivin is periodically expressed during the cell cycle peaking in mitosis [8]. Previou studies have shown that Survivin expression is impaired by p53, and loss of p53 function, which is often observed in cancer cells, increases its transcription [9], [10]. Indeed most cancer cells analyzed so far up-regulate Survivin [11], [12] when compared to normal cells or adjacent tissues. In addition, in many human cancer cases, Survivin has been detected even during interphase [13], [14]. Overexpression of Survivin is frequently found in colorectal cancer [15], [16], [17]. Here increased levels of Survivin have been described to correlate with a poor prognosis [18], [19], but contradictory data have been published regarding this [20]. Besides its CPC functions Survivin is thought to have multiple roles in apoptotic regulation [21], [22], [23]. However, within the N-terminus of Survivin, there is only one baculoviral inhibitor of apoptosis repeat (BIR) domain. Also Survivin contains no C-terminal RING finger domain which is common for members of the IAP family [11], [24]. Although Survivin was originally classified as inhibitor of apoptosis (BIR) it is now recognized that its main molecular functions are linked to the spindle assembly checkpoint and cytokinesis. Nowadays, the anti-apoptotic functions of Survivin are questioned since most experiments which blocked Survivin function led to the development of mitotic defects which might interfere with an accurate analysis of its IAP function [25], [26], [27], [28]. In a previous study ablation of Survivin in chicken B lymphocyte DT40 was lethal because of the lack of both CPC functions and additional other cell proliferative abilities [29]. Yet, these cells displayed normal susceptibility to etoposide-induced apoptosis when compared to control cells.

In our study we show for the first time that detergent-soluble Survivin suppresses anoikis in anchorage-dependent cells, a form of programmed cell death induced by loss of adhesion from the surrounding extracellular matrix. Subcellular fractionation experiments provided evidence that detergent soluble Survivin of the cytosol was responsible for suppressing anoikis. The mechanism of anoikis suppression involves activation of XIAP/IκB-α/NF-κB signaling and inactivation of c-Jun. A further important finding of this study is that detergent-soluble Survivin is correlated to the invasive phenotype of colorectal cancer cells. Therefore, detergent-soluble Survivin might be of clinical value since it likely predicts lymph node and distant metastases in colorectal cancer patients.

## Results

### Overexpression of Survivin protects cells from anoikis

**Figure 1:**
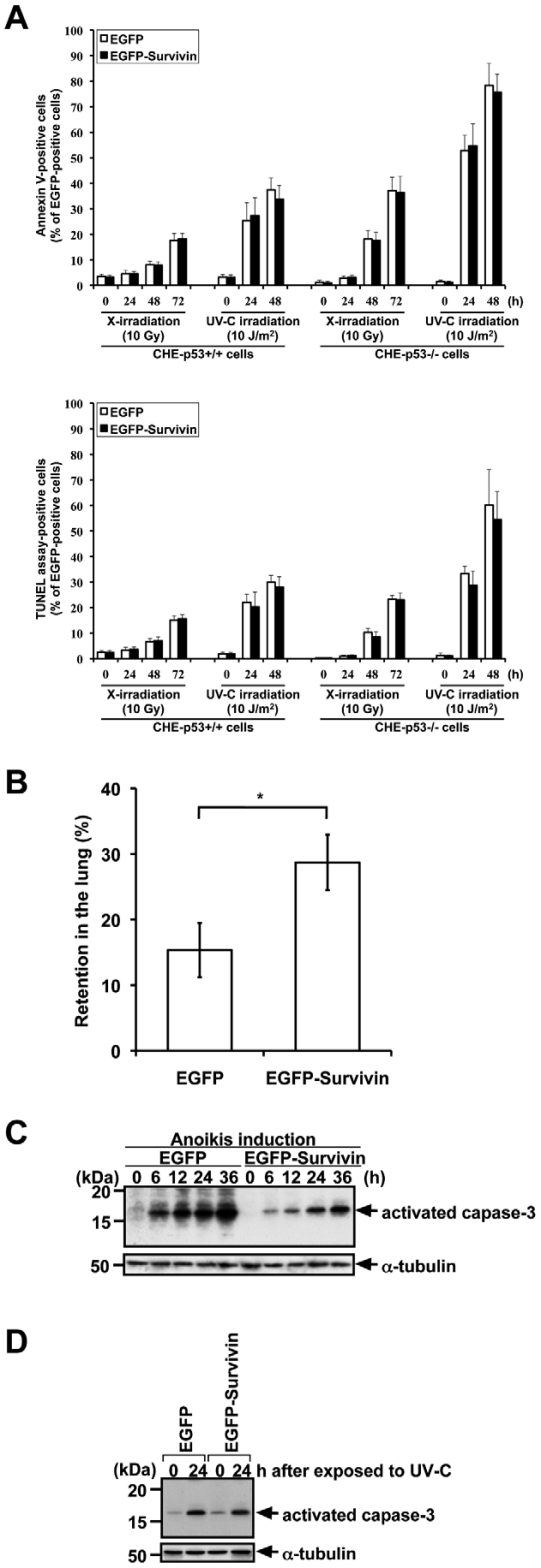
Overexpression of Survivin does not significantly protect radiations-induced apoptosis, but does protect apoptosis under other stresses. **A.** Frequency of apoptosis in CHE cells (with p53+/+ and p53−/−) transfected with pEGFP-empty and pEGFP-Survivin after treatment with X-irradiation (10 Gy) and UV-C (10 J/m^2^). The transfected cells were exposed to IR or UV-C at 24 h after transfection. Transfection frequencies were 80–90%, and EGFP-positive cells were counted for Annexin V-positive or -negative cells (*upper panel*) and TUNEL assay-positive or -negative cells (*lower panel*). X-irradiated CHE-p53−/− cells had significantly greater fraction of apoptosis-positive cells when compared to X-irradiated CHE-p53+/+ cells (*P*<0.03 for Annexin V staining and *P*<0.08 for TUNEL assay), and UV-C irradiated CHE-p53−/− cells had significantly greater fraction of apoptosis-positive cells when compared to UV-C irradiated CHE-p53+/+ cells (*P*<0.001 for Annexin V staining and *P*<0.02 for TUNEL assay). Apoptosis-positive cells were not significantly decreased in pEGFP-Survivin-transfected cells when compared to pEGFP-transfected cells in each case (*P*>0.4 for Annexin V staining and *P*>0.4 for TUNEL assay). Values indicate means ± S.D. (n = 3). **B.** Increase of the retention in the lung after intravenous injection of EGFP- or EGFP-Survivin-expressing cells. The number of surviving CHE-p53−/− cells in the lung was significantly increased when compared to the number of surviving control cells expressing EGFP. Values indicate means ± S.D. of six mice. *Significant difference (*P*<0.005). **C.** Caspase-3 activation in CHE-p53−/− cells transfected with pEGFP-empty and pEGFP-Survivin after anoikis induction. The transfected cells were detached from extracellular matrix and simultaneously serum-starved to induce anoikis at 24 h after transfection. Cells were suspended in serum-free medium for 6–36 h, harvested, and lysed in Laemmli SDS-sample buffer for immunoblot analysis with anti-activated caspase-3 antibody. Transfection frequencies were checked by using fluorescence microscopy and confirmed to be 80–90%. **D.** Caspase-3 activation in CHE-p53−/− cells transfected with pEGFP-empty and pEGFP-Survivin after treatment with UV-C (10 J/m^2^). The transfected cells were exposed to UV-C at 24 h after transfection. Cells were cultured for 24 h, harvested, and lysed in Laemmli SDS-sample buffer for immunoblot analysis with anti-activated caspase-3 antibody. Transfection frequencies were checked by using fluorescence microscopy and confirmed to be 80–90%.

In initial studies we sought to analyze the impact of Survivin overexpression in p53-deficient Chinese hamster embryonic diploid fibroblasts (CHE cells) and isogenic CHE cells harboring wild type p53. For monitoring transfection we used an enhanced green fluorescence protein-tagged Survivin (EGFP-Survivin). Yet, the ectopic overexpression of EGFP-Survivin had no effect on apoptosis in CHE-p53+/+ cells treated with DNA damaging ionizing radiation (IR) and UV-C when compared to CHE-p53+/+-EGFP cells (Figure 1A) as measured by annexin V staining and terminal deoxynucleotidyl transferase (TdT)-mediated dUTP nick end labeling (TUNEL) assay. Then we turned our attention to CHE cells with defective p53 status (CHE-p53−/− cells). Strikingly, CHE-p53−/− cells with ectopic overexpression of EGFP-Survivin produced metastatic lung tumors when injected subcutaneously into immuno-deficient mice whereas no lung metastases were observed after xenografting CHE-p53−/− cells expressing EGFP (Table S1). Testing apoptosis induction after IR or UV-C revealed that CHE-p53−/− cells had significantly greater fraction of apoptosis-positive cells when compared to CHE cells having wild type p53 [30], [31], [32] (Figure 1A). This observation is in line with reports using rodent embryonic fibroblasts demonstrating a less susceptibility to IR and UV-C than those cells which lack functional p53, cell cycle checkpoint control. Interestingly, we also did not find a protective effect of overexpressed Survivin in CHE-p53−/− cells when compared to CHE cells with ectopic expression of EGFP (Figure 1A).

So far transfection of pEGFP-Survivin does not inhibit radiation–induced apoptosis in CHE-cells, irrespective of p53 status and expression levels of Survivin but on the other hand only CHE-p53−/− cells overexpressing EGFP-Survivin caused metastatic disease in mice. We therefore hypothesized that overexpression of Survivin might increase the survival of CHE-p53−/− -EGFP-Survivin cells under stress conditions which likely more resembles the *in vivo* physiological conditions of disseminating tumor cells such as anchorage-independent situation and nutrient starvation.

Indeed, by applying *i.v.* injection of tumor cells in mice we revealed that the number of surviving CHE-p53−/− cells in the lung was significantly increased when compared to the number of surviving control cells expressing EGFP (Figure 1B).

**Figure 2:**
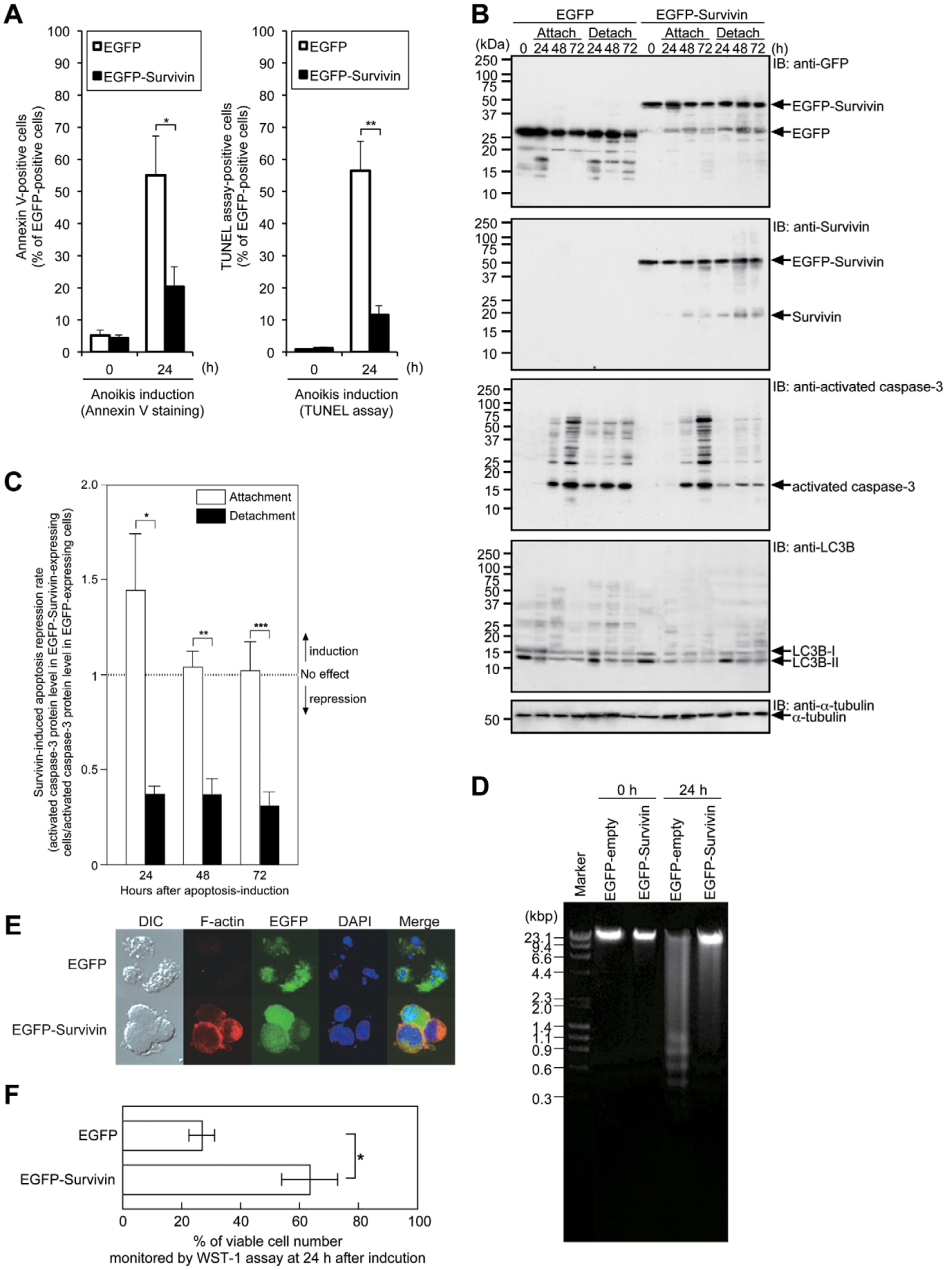
Overexpression of Survivin predominantly protects anoikis. **A.** Frequency of anoikis in CHE-p53−/− cells transfected with pEGFP-empty and pEGFP-Survivin. The transfected cells were detached from extracellular matrix and simultaneously serum-starved to induce anoikis at 24 h after transfection. Transfection frequencies were 80–90%, and EGFP-positive cells were counted for anoikis-positive or -negative cells. Overexpression of EGFP-Survivin significantly suppressed anoikis when compared to the CHE-p53−/− control cells. Values indicate means ± S.D. (n = 3). *Significant difference (*P*<0.08). **Significant difference (*P*<0.04). **B.** Caspase-3 activation in EGFP- and EGFP-Survivin-expressing cells after serum starvation under attached or detached culture conditions. The experimental protocol was illustrated in Figure S1. Transfection frequencies were checked by using fluorescence microscopy and confirmed to be 80–90%. Cells were kept in serum-free medium for 24–72 h, harvested, and lysed in Laemmli SDS-sample buffer for immunoblot analysis with anti-GFP, anti-Survivin, anti-activated caspase-3 antibody, anti-LC3B, and anti-α-tubulin. **C.** Survivin-induced apoptosis repression rate compared between EGFP-expressing cells and EGFP-Survivin-expressing cells. Levels of activated caspase-3 protein were estimated by immunoblot analysis. The repression rates were expressed in a ratio of activated caspase-3 protein level in EGFP-Survivin-expressing cells to the level in EGFP-expressing cells. Values indicate means ± S.D. (n = 3). *Significant difference (*P*<0.04). **Significant difference (*P*<0.02). ***Significant difference (*P*<0.007). **D.** DNA fragmentation analysis in EGFP-expressing cells and EGFP-Survivin-expressing cells. The transfected cells were detached from extracellular matrix and simultaneously serum-starved to induce anoikis at 24 h after transfection. The cells were suspended for 24 h, and harvested. Genomic DNA was extracted and electrophoresed on agarose gels. **E.** Representative images of EGFP-expressing cells and EGFP-Survivin-expressing cells, using laser confocal microscopy. Transfected cells were suspended in serum-free medium for 24, and imaged by Nomarski differential interference contrast (*lane DIC*), by rhodamine phalloidin staining (*lane F-actin*), by EGFP fluorescence (*lane EGFP*), and by DAPI staining (*lane DAPI*). Rhodamine phalloidin staining and DAPI staining typically indicated that EGFP-expressing cells underwent anoikis, but EGFP-Survivin-expressing cells did not. **F.** Determination of cell viability by WST-1 assay. Transfected cells were suspended in serum-free medium for 24 h, and then cell viability of EGFP-expressing cells and EGFP-Survivin-expressing cells was assessed. Values indicate means ± S.D. (n = 3). *Significant difference (*P*<0.04).

To further explore our hypothesis, we tested whether overexpression of EGFP-Survivin conferred reduced anoikis-susceptibility, namely resistance to serum starvation- and suspension culture-induced apoptosis, in CHE cells. As depicted in Figure 2A it became obvious that overexpression of EGFP-Survivin significantly suppressed anoikis when compared to the CHE-p53−/− control cells, as measured by annexin V staining and also by TUNEL assay, in CHE-p53−/− cells. Additional immunoblot analysis for detection of the processed executor caspase-3 confirmed that overexpressed EGFP-Survivin protected from anoikis (Figure 1C) since CHE-p53−/− cells with ectopic expression of EGFP-Survivin demonstrated a weaker gradual increase of processed caspase-3 during the observed period of time. On the other hand, overexpression of EGFP-Survivin did not suppress caspase-3 activation in UV-C-induced apoptosis (Figure 1D).

Next, we determined whether the protective effect of overexpressed EGFP-Survivin on caspase-3 activation was anoikis-specific, by using an anoikis assay protocol (schematically illustrated in Figure S1). Immunoblot analyses showed that the suppressive effect was more potent in detached culture condition than in attached culture condition (Figure 2B). Noteworthy, autophagy, another type of cell death caused by a catabolic process for the degradation of cellular components by lysosome, was not involved in the effect (Figure 2B, *IB: anti-LC3B*). The quantitative data demonstrated the effective suppression of suspension culture-induced anoikis in EGFP-Survivin-expressing cells when compared to control cells expressing EGFP (Figure 2C). In line with this observation, overexpression of EGFP-Survivin suppressed DNA fragmentation (Figure 2D and E) and preserved cell viability (Figure 2F) in detached culture condition.

### Overexpression of Survivin regulates apoptosis-related transcription factors

**Figure 3:**
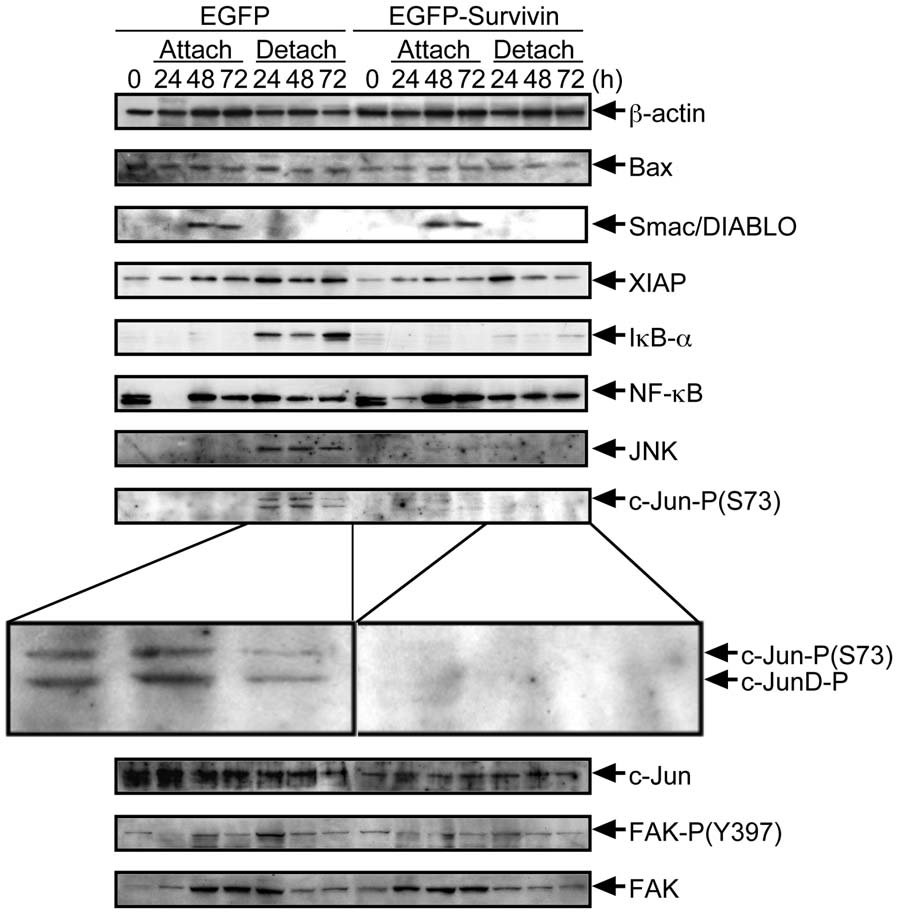
Expression and phosphorylation of signaling molecules relating to Survivin in EGFP- and EGFP-Survivin-expressing cells after serum starvation under attached or detached culture conditions. The experimental protocol was illustrated in Figure S1. Transfection frequencies were checked by using fluorescence microscopy and confirmed to be 80–90%. Cells were kept in serum-free medium for 24–72 h, harvested, and lysed in Laemmli SDS-sample buffer for immunoblot analysis with anti-β-actin, anti-Bax, anti-Smac/DIABLO, anti-XIAP, anti-IκB-α, anti-NF-κB, anti-JNK, anti-c-Jun-P(S73), anti-c-Jun, anti-FAK-P(Y397), and anti-FAK.

We next asked which signaling pathways could be involved in the anoikis-suppression in EGFP-Survivin-expressing CHE-p53−/− cells. Survivin has been described to inhibit pro-apoptotic Bcl-2-associated X protein (BAX)-induced apoptosis [33]. Yet expression levels of BAX were not altered after induction of anoikis in EGFP-Survivin- as well as EGFP-expressing CHE-p53−/− cells (Figure 3, *Bax*). We then focused on the second mitochondria-derived activator of caspase/direct inhibitor of apoptosis-binding protein with low pI (Smac/DIABLO) which has been demonstrated to bind to Survivin [34], [35]. However, Smac/DIABLO was undetectable in detached culture condition (Figure 3, *Smac/DIABLO*). Another IAP family member, the X-linked inhibitor of apoptosis protein (XIAP) has been reported to form heterodimers with Survivin [36]. Some lines of evidence suggest that this heterodimer is able to activate an anti-apoptotic NF-κB signaling, via phosphorylation and subsequent degradation of the molecular inhibitor of kappa B-α (IκB-α) [37]. Interestingly, we noted an upregulation of IκB-α protein levels in detached culture condition of control CHE-p53−/− cells whereas IκB-α expression levels remained very low in EGFP-Survivin-expressing cells (Figure 3, *XIAP, IκB-α, and NF-κB*). Concomitantly, augmentation of phosphorylation of pro-apoptotic transcription factor, c-Jun, was suppressed by overexpression of EGFP-Survivin in detached culture condition (Figure 3, *JNK, c-Jun-P(S73), and c-Jun*). These data suggest that overexpression of EGFP-Survivin decreases the IκB-α level in detached cells and subsequently lead to the activation NF-κB and on the same time to a suppression of c-Jun-mediated transcription. However, we could not found a contribution of focal adhesion kinase (FAK) to anoikis suppression in this cell system although FAK auto-phosphorylation site at Y397 has been reported to be important for anti-apoptotic activity of FAK [38], [39].

**Figure 4:**
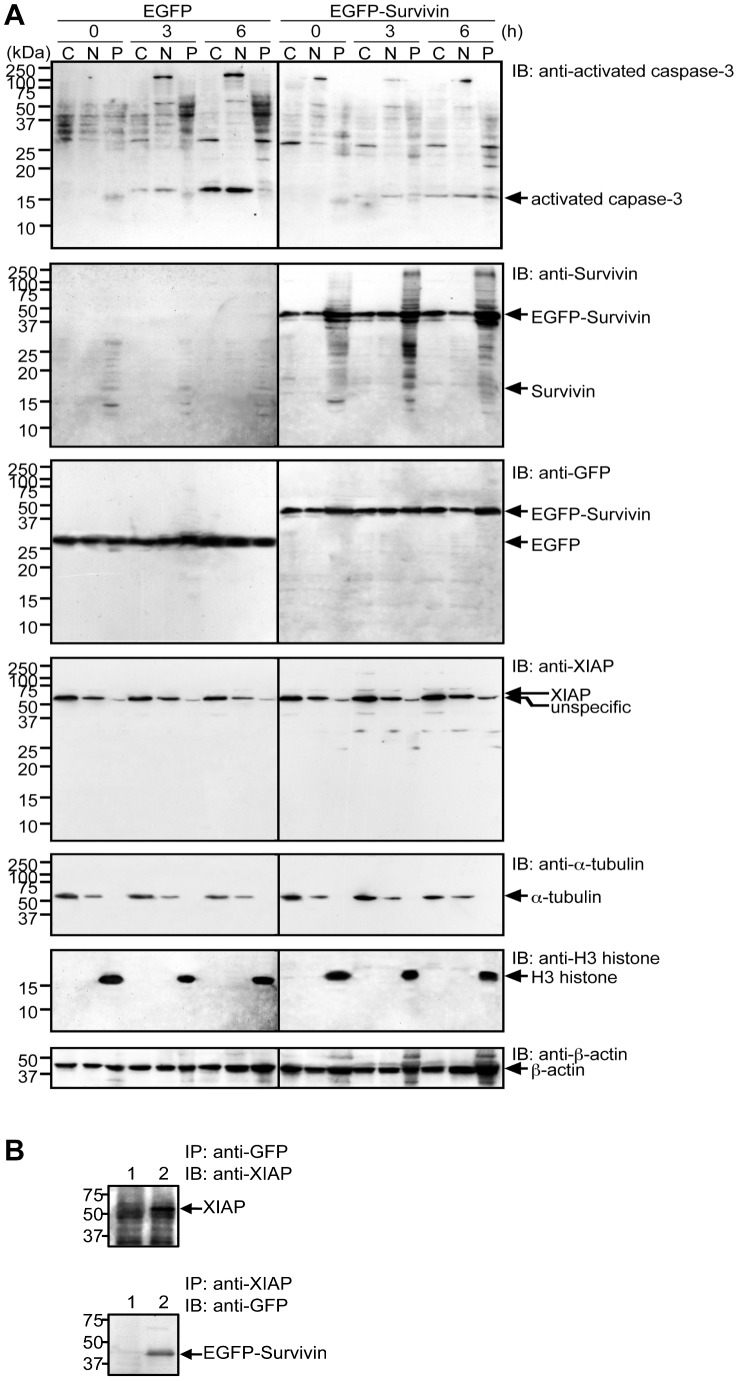
EGFP-Survivin and XIPA found in the detergent-soluble fractions. **A.** Subcellular fractionation and immunoblot analysis of activated caspase-3, Survivin, and XIAP. Transfection frequencies were checked by using fluorescence microscopy and confirmed to be 80–90%. Cells were kept in serum-free medium for 3–6 h, harvested, and lysed. The cell lysate was fractionated into the detergent-soluble cytoplasmic (*C*), and nuclear (*N*) fractions and the detergent-insoluble pellet (*P*) fraction for immunoblot analysis with anti-activated caspase-3, anti-Survivin, anti-GFP, anti-XIAP, anti-α-tubulin, anti-H3-histone, and anti-β-actin. **B.** Protein-protein interactions between EGFP-Survivn and XIAP. Cell lysate from the detergent-soluble cytoplasmic fraction was immunoprecipitated by anti-XIAP antibody and anti-GFP antibody and subsequently immunoblotted with anti-GFP antibody (*lower panel*) and anti-XIAP antibody (*upper panel*), respectively. EGFP-Survivin and XIAP were co-imunoprecipitated only in cell lysate form EGFP-Survivin-expressing cells (*lane 2*).

CPC components including Survivin have a tendency to associate with chromatin and therefore are often found in the nucleus and are mostly detergent-insoluble. Yet, other intracellular pool of Survivin has been described in the cytosol or associated with mitochondria [40], [41]. It was of special interest how Survivin associates with XIAP in a cell. From time-course experiments, caspase-3 activation was found 6 h after anoikis induction (Figure 1C). Accordingly, the cells at time 0, 3, and 6 h following anoikis induction were used for subcellular fractionation experiments to avoid misinterpretations that can arise from using dead cells induced by anoikis. Immunoblot analyses following subcellular fractionation revealed that XIAP was mostly found in the detergent-soluble fraction of the cytosol (Figure 4A, *IB: anti-XIAP*). Yet, the majority of EGFP-Survivin was found in the detergent-insoluble pellet fraction containing chromatin but was also found to a lesser extend in the detergent-soluble nuclear and cytosolic fractions (Figure 4A, *IB: anti-Survivin and IB: anti-EGFP*). Thus, an interaction of XIAP and Survivin obviously was restricted to the detergent soluble fraction of the cytosol. Indeed we found a stable protein-protein interaction between XIAP and Survivin by applying immunoprecipitation experiments using the detergent-soluble cytoplasmic fraction of CHE-p53−/− cells with ectopic expression of EGFP-Survivin (Figure 4B). In addition, non-tagged Survivin was less effectively fractionated into the detergent-soluble cytoplasmic fraction and anoikis and caspase-3 activation were also less effectively suppressed in non-tagged Survivin-overexpressed CHE-p53−/− cells. On the other hand, a Discosoma red fluorescent protein-tagged Survivin (Survivin-DsRed) was more effectively fractionated into the detergent-soluble cytoplasmic fraction and anoikis and caspase-3 were also more effectively suppressed in Survivin-DsRed-overexpressed CHE-p53−/− cells (our unpublished data).

### The Detergent-soluble Cytoplasmic Survivin is involved in metastatic progression of human colorectal cancer

**Figure 5:**
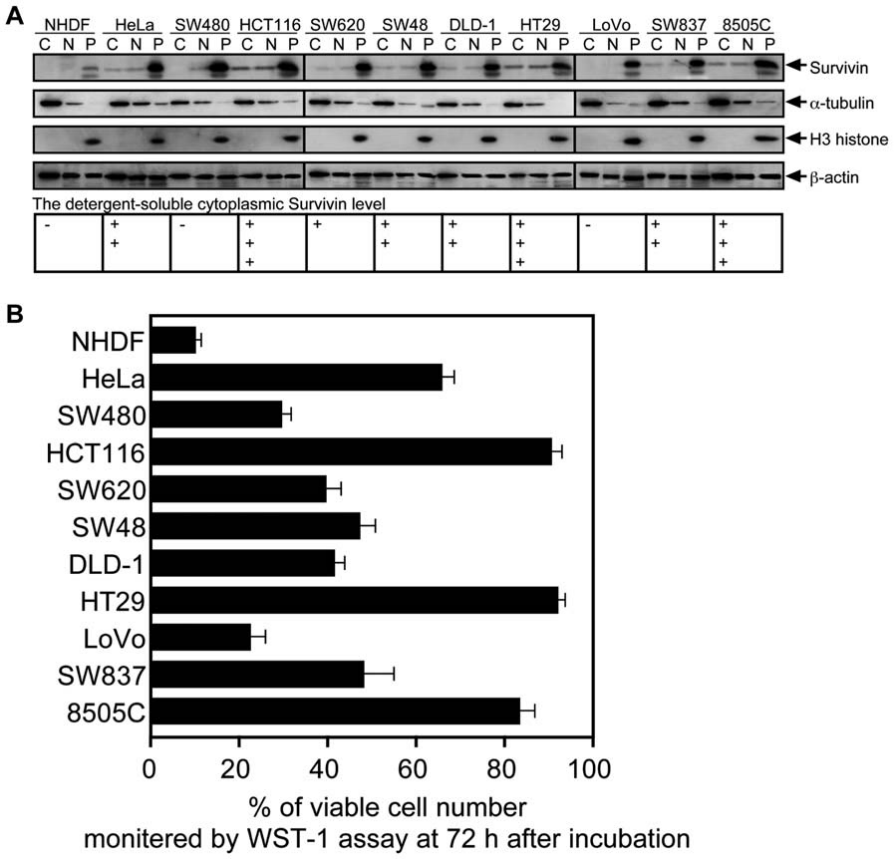
Survivin expression and anoikis susceptibility in colorectal cancer cells. **A.** Subcellular fractionation and immunoblot analysis of Survivin. The cell lysate from exponentially growing cells was fractionated into the detergent-soluble cytoplasmic (*C*), and nuclear (*N*) fractions and the detergent-insoluble pellet (*P*) fraction for immunoblot analysis with anti-Survivin, anti-α-tubulin, anti-H3-histone, and anti-β-actin. The detergent-soluble cytoplasmic Survivin level was estimated by immunoblot analysis, and expressed as a percentage of the level of 8505C, which is undifferentiated thyroid carcinoma showing anoikis resistance (+++, >80%; ++, 40–80%; +, 10–40%; −, <10%). **B.** Anoikis susceptibility in in colorectal cancer cells. Cells were suspended in serum-free medium for 72 h. Cell viability was determined by WST-1 assay.

We finally addressed the question whether detergent-soluble cytoplasmic Survivin, is found in human cancer and is somehow linked to the development of metastatic disease. We analyzed eight colorectal cancer cell lines, the HeLa cervix carcinoma cell line, and the 8505C throid carcinoma cell line by cellular fractionation and immunoblotting. As control we included normal embryonic diploid fibroblast (NHDF). Among the colorectal cancer cell lines, Survivin was found in the detergent-soluble fractions although the expression levels varied considerably (Figure 5A). Subsequently, anoikis-susceptibility was examined in all cancer cell lines (Figure 5B). Although the magnitude of the contribution of Survivin to anoikis resistant phenotype in colorectal cancer cells is undefined, there is a direct correlation between higher expression levels of the detergent-soluble cytoplasmic Survivin and anoikis resistance in particular for HCT116 and HT29 cells with highest expression of detergent soluble fraction of the cytoplasm. Noteworthy, NHDF, LoVo and SW480 cells, in which detergent-soluble Survivin was not detectable, showed massive cell death after induction of anoikis.

**Figure 6:**
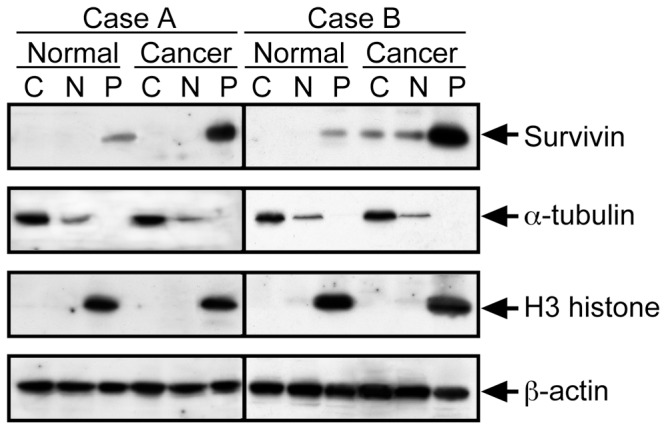
Representative data for subcellular fractionation and immunoblot analysis of Survivin in clinical samples. The oultline of the method was illustrated in Figure S6. Normal and Cancerous tissues from human colorectal cancer patients without metastases (*Case A*) or with metastases (*Case B*) were lysed. The cell lysate was fractionated into the detergent-soluble cytoplasmic (*C*), and nuclear (*N*) fractions and the detergent-insoluble pellet (*P*) fraction for immunoblot analysis with anti-Survivin, anti-α-tubulin, anti-H3-histone, and anti-β-actin.

Which biological behaviors in colorectal cancer are related to detergent-soluble cytoplasmic Survivin is important for its diagnostic or therapeutic availabilities. In our previous immunohistochemical analyses of colorectal cancer tissues, the absence of nuclear Survivin and the existence of cytoplasmic Survivin have found to be significant predictors of mortality in colorectal cancer patients [42]. Yet, the analysis of the immunohistochemical localization and the subcellular fractionation detection are not the same. Five samples of normal and corresponding primary cancerous tissues from the same patients were examined by subcellular fractionation experiments, but regardless of the levels of Survivin overexpression, the cases positive for the detection of the detergent-soluble cytoplasmic Survivin were patients with lymph node and other distant metastases. Here, other forty cases were examined. The outline of the detection method was illustrated in Figure S2. Figure 6 shows a typical results from two patients with or without the detergent-soluble cytoplasmic Survivin in primary cancerous tissues (case A is a patient without metastasis and case B is a patient with metastasis). Clinicopathological significance of the detergent-soluble cytoplasmic Survivin expression was determined for the forty cases. The positive cases were significantly increased in tumor size (<0.005), primary colorectal cancer with lymph node metastasis (<0.001), and primary colorectal cancer with distant metastasis (<0.01), compared with the negative cases (Table 1).

**Table 1:** Clinicopathological significance of the detergent-soluble cytoplasmic Survivin expression in colorectal cancer.

Factors	Tumor without the detergent-soluble cytoplasmic Survivin (n = 23)		Tumor with the detergent-soluble cytoplasmic Survivin (n = 17)		*P* value
	No.	%	No.	%	
Age (mean ± SD)	65.2±10.9	65.0±8.9			
Sex					
Male	17	73.9	8	47.1	0.08
Female	6	26.1	9	52.9	
Histological grade					
Well, Moderately	19	82.6	13	76.5	0.46
Poorly, Muc expression	4	17.4	4	23.5	
Size					
<50 mm (small)	12	70.1	3	30	<0.05*
>51 mm (large)	5	29.9	7	70	
Tumor invasion^a^					
m, sm, mp, ss	19	82.6	10	58.8	0.095
se, si	4	17.4	7	41.2	
Lymphatic invasion					
Absent	15	65.2	6	35.3	0.061
Present	8	34.8	11	64.7	
Venous invasion					
Absent	20	87.0	11	64.7	0.100
Present	3	13.0	6	35.3	
Lymph node metastasis					
Absent	16	69.6	1	5.9	<0.001*
Present	7	30.4	16	94.1	
Distant metastasis					
Absent	23	100	12	70.6	<0.01*
Present	0	0	5	29.4	

a Tumor invasion of mucosa (m), submucosa (sm), muscularis propria (mp), subserosa (ss), penetration of serosa (se), and invasion of adjacent strucures (si).

* Significant difference.

## Discussion

Nowadays, it is unanimously accepted that Survivin is an essential component of the CPC regulating chromomal segregation and cytokinesis [2] although the exact roles of Survivin in mitosis are still not fully understood. Cells with impaired function of Survivin or of one of its partners due to RNAi-mediated inhibition or expression of dominant-negative mutants showed comparable phenotypes (i.e., disturbed segregation of chromosomes and defective cytokinesis) [25], [26], [28], [43], [44], [45]. Furthermore, the phenotypes of Survivin knock-out mutants in yeasts [46], [47] and *C. elegans* [48], [49] confirmed the role of Survivin as CPP. In vertebrates, the essential role of Survivin during mitosis has been demonstrated in *Xenopus laevis* [3] and mice [50]. On the other hand, various studies revealed an anti-apoptotic function of Survivin in different species and cell lines. Anti-apoptotic effects of Survivin and of the Survivin-like protein Deterin were reported in yeast [51] and *D. melanogaster* [52], respectively. Yet, overexpression of Survivin might act as an anti-apoptotic factor even in non-vertebrates under certain conditions, but it appears conceivable that the Survivin-knockout phenotypes which were interpreted as loss of anti-apoptotic function might be primarily linked to deregulated mitotic processes.

In cultured cell systems, an increased apoptotic susceptibility, which appeared spontaneously or by apoptotic agents, is conferred by loss-of-function or knock-down of Survivin. Survivin knock-out in a T-cell lineage induced p53-dependent phenotypes, resulting in thymocyte developmental defect [53]. This phenotype could not be rescued by inactivation of p53, suggesting that Survivin is likely to be an anti-apoptotic factor, regardless of p53 status. In normal human fibroblasts, Survivin knock-down also induced p53 induction, which triggered cell cycle arrest without immediate apoptosis [54]. This phenotype was rescued by inactivation of p53, suggesting that Survivin is likely to work via p53 under adherent culture condition. These different results are obscuring information for understanding Survivin-induced protection from apoptosis. The different cell types, adherent cells and non-adherent cells, are different in p53-induced phenotypes. Non-adherent cells with normal p53 predispose to cellular stress-induced immediate apoptosis [55], so-called interphase cell death, compared with adherent cells, while adherent cells with p53 primarily arrest the cell cycle [56], [57]. Survivin knock-out induces p53 in both adherent and non-adherent cells, consequently expressing the phenotypes for each type of the cells. In our hypothesis which is supported by our study, Survivin is preferentially inhibiting anoikis in cells subjected to anchorage-dependent survival and growth irrespective of p53 status.

When exposed to the DNA damaging agent etoposide, Survivin knocked-out DT40 cells showed normal sensitivity to this agent [29]. The data suggested that Survivin is not a universal inhibitor for DNA-damaging agent-induced apoptosis. Here we observed normal sensitivities to IR and UV-C in Survivin-overexpressing CHE-p53−/− cells. In our view, Survivin would have important roles in anoikis suppression for cancer development (see below).

**Figure 7:**
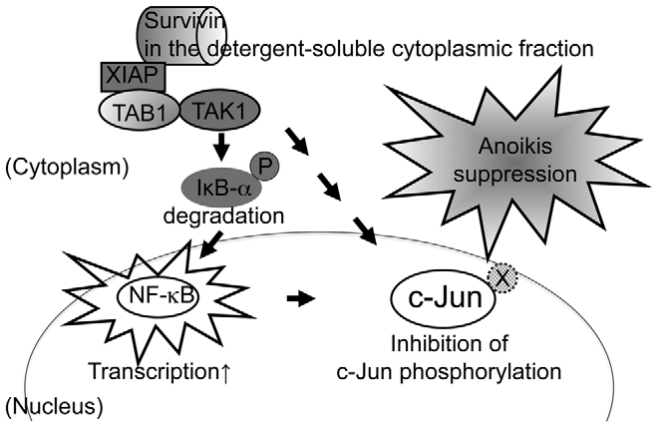
Anoikis suppression pathways triggered by the detergent-soluble cytoplasmic Survivin. Overexpressed Survivin, which is fractionated into the detergent-soluble cytoplasmic fraction, associates with XIPA. The Survivin-XIPA heterodimer increases NF-κB transcriptional activity due to IκB-α degradation and NF-κB nuclear translocation. Simultaneously, the Survivin-XIPA heterodimer inhibits c-Jun phosphorylation. In our proposed model, cross-talk between the NF-κB and JNK pathways relies on the Survivin-XIPA heterodimer leading to the shutdown of JNK activity and to rescue from anoikis.

In line with previous reports, we show here that Survivin regulates caspase-3 activity. It has been reported that Survivin inhibits caspase-3 activation in physiological situations during apoptosis, but this effect is likely not due to the direct inhibition of caspase-3 [58], [59], [60]. Hence the precise anti-apoptotic mechanism of Survivin still remains a challenge. Two mechanisms have been proposed to account for the inhibition of mitochondria-regulated apoptosis resulting in caspase-3 activation. The most studied molecule for this is a pro-apoptotic protein Smac/DIABLO, to which Survivin binds directly [34]. A Smac/DIABLO-mediated interaction with Survivin to suppress anoikis appears unlikely since in our experimental setting only little or no expression of Smac/DIABLO during anoikis of CHE-cells was detected (Figure 3). The second mechanism how Survivin fulfills its IAP function is a direct binding to XIAP [36]. Current evidence suggests that anti-apoptosis is achieved by Survivin/XIAP heterodimer complex in two ways. In the first way, the binding of Survivin to XIAP enhances XIAP stability by preventing ubiquitin-mediated proteolysis [36], [61]. XIAP has a C-terminal RING finger domain, by which caspase-3 is thought to be directly down-regulated [62], [63], [64]. In the second way, Survivin/XIAP participates in activation of XIAP/IκB-α/NF-κB signaling [37]. Downregulation of IκB-α is critical for transcription factor NF-κB activation [65], [66]. Indeed in our study, we found that overexpression of Survivin activated this pathway and that simultaneously c-Jun was inactivated for suppression of anoikis (Figure 7).

The detergent-soluble cytoplasmic Survivin was important to induce anoikis suppression in our experimental system. There is a proposal that Survivin releases from mitochondria in response to cell stresses, resulting in Survivin/XIAP heterodimer formation under the regulation by phosphorylation of Survivin at serine 20 [24]. This phosphorylation site is human-specific (alanine for mouse), suggesting another mechanism underlying this regulation. In addition, monomeric Survivin is suggested to be important for apoptosis regulation [67].

**Figure 8:**
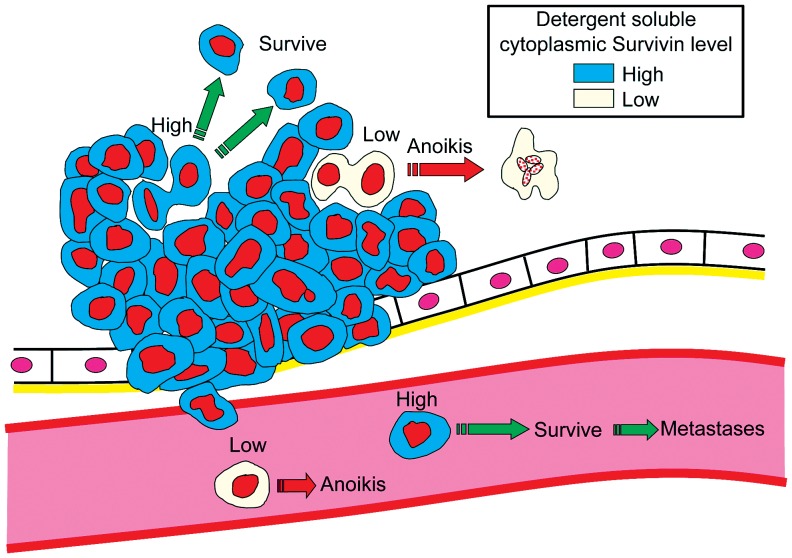
Survivin functions during metastases. The detergent soluble cytoplasmic Survivin is imperative for survival of not only primary tumor cells but also intravasated cells.

The regional lymph node metastasis as well as the distant metastasis are well recognized in colorectal cancer staging and are known to be a common determinant of long-term outcome in the cancer patients. The metastasis-related factors, genes, or proteins, which have the prognostic significance and clinical impact in patients with colorectal cancer, still need identifying. The information promises to advance the understanding, treatment, and prevention of colorectal cancer. The detergent-soluble cytoplasmic Survivin is a candidate such a factor from our analysis. Anoikis resistance is a key for cancer cell survival not only in the primary tumor microenvironment but also for invaded and extravasated cancer cells (Figure 8). Our data suggests that Survivin plays a critical role in cancer cell fate in a body, supporting recent developments of small Survivin inhibitor for molecular targeting therapy [68].

## Materials and Methods

### Cell lines and cell culture

CHE cells were isolated from Chinese hamster whole embryos during *in vitro* cell transformation assay [56]. Clone A1/p60/clone #4 with a normal modal chromosome number of 22 having normal *p53* were used as CHE-p53+/+ cells, and clone A1/p60/clone #3 with a modal chromosome number of 23 containing one t(1q;9) marker chromosome having mutated *p53* at codon 245 (GGC/AGC) in both alleles were used as CHE-p53−/− cells. CHE-p53−/− cells are non-metastatic when injected subcutaneously or intravenously into nude mice, but become metastatic by introducing certain metastasis-relating genes [57]. HeLa cells and colorectal cancer cells were obtained from American Type Culture Collection, and thyroic cancer cells 8505C was obtained from RIKEN BioResource Center. Normal embryonic diploid fibroblast (NHDF) cells were obtained from Kurabo Industries Japan. All cells were cultured in Dulbecco's modified minimal essential medium supplemented with 10% fetal bovine serum in 5% CO_2_ at 37°C.

### Protein fractionation and Immunoblot analysis

Whole cell lysates were prepared by lysed with Laemmli SDS-PAGE sample buffer. Nuclear and cytoplasmic extracts from cultured cells or human tissues were prepared using a Nuclear Extraction Kit (Active Motif), according to the manufacturer's instructions. The pellet was used as the detergent-insoluble fraction after solubilization by Laemmli SDS-PAGE sample buffer. This fraction contains detergent-insoluble cytoplasmic and nuclear fractions. SDS-PAGE and immunoblotting were performed according to the procedures described previously [55]. Anti-GFP antibody (JL-8, Clontech Laboratories), anti-Survivin antibody (NB 500-201, Novus Biologicals; NB 500-237, Novus Biologicals; sc-10811, Santa Cruz Biotechnology), anti-activated caspase-3 antibody (#9661, Cell Signaling Technology), anti-LC3B antibody (#4445, Cell Signaling Technology), anti-α-tubulin antibody (CLT9002, Cedarlane Laboratories), anti-β-actin antibody (A1978, Sigma-Aldrich), anti-Bax antibody (sc-493, Santa Cruz Biotechnology), anti-Smac/DIABLO antibody (PM004, MBL International), anti-XIAP antibody (AF822, R&D Systems), anti-IκB-α antibody (sc-371, Santa Cruz Biotechnology), anti-NF-κB antibody (sc-372, Santa Cruz Biotechnology), anti-JNK antibody (sc-474, Santa Cruz Biotechnology), anti-phosphorylated c-Jun at serine 73 antibody (#06-659, EMD Millipore), anti-c-Jun antibody (#09-754, EMD Millipore), anti-phosphorylated FAK at tyrosine 397 antibody (F25420, BD Biosciences), and anti-FAK antibody (sc-932, Santa Cruz Biotechnology) were used for Immunoblot analysis.

The activated caspase-3-specific bands were quantitatively measured by a fluorescence imaging system (ImageQuant LAS, GE Healthcare Life Sciences) using immnoblots developed by ECF Western Blotting Reagent Pack (GE Healthcare Life Sciences). The protein levels of activated caspase-3 were calculated by the following formula: (estimated activated caspase-3 level) = (immunofluorescence intensity of activated caspase-3-specific band)/(immunofluorescence intensity of α-tubulin-specific band on the same blot).

### Apoptosis and anoikis assays

Cells were transfected with pEGFP (#6084-1, BD Biosciences) or pEGFP-Survivin (pEGFP with the full-length mouse Survivin cDNA) by using Lipofectamine 2000 (Life Technologies). The transfected cells were exposed to serum-starvation at 24 h after transfection. For anoikis induction, transfected cells were suspended in serum-free medium (Figure S1). Apoptosis (or anoikis) was determined by annexin V-Alexa 568 (Roche Applied Scicence) staining, and also confirmed by TUNEL assay using TMR red (Roche Applied Scicence). Transfection frequencies were 80–90%, and EGFP-positive cells were counted for apoptosis (or anoikis)-positive or -negative cells.

DNA fragmentation analysis was performed as described [69]. Cell viability was assessed by tetrazolium salt (WST-1) assay using Cell Proliferation Reagent (Roche Applied Science).

### Indirect immunofluorescence

Transfected cells were fixed with 4% formaldehyde-containing Formaldehyde Neutral Buffer Solution (Nakarai Tesque). The fixed cells were then stained with 200 U/ml rhodamine phalloidin (Molecular Probes) plus 0.1 µg/ml 4′,6-Diamidino-2-phenylindole (DAPI), mounted onto an anti-fade fluorescent mounting medium (DakoCytomation), and observed under a FV1000D laser scanning microscope (Olympus).

### Immunoprecipitation analysis

The detergent-soluble cytoplasmic fraction was used for Immunoprecipitation analysis. The cleaned extract was incubated with affinity-purified Rabbit anti-XIAP polyclonal antibody coupled to protein A Dynabeads (Life Technologies). The beads were washed and were processed for immunoblot analysis with monoclonal anti-GFP antibody. Immunoprecipitation of GFP-Survivin was carried out under the same conditions using anti-XIAP antibody.

### Assay of retention of tumor cells in the lung

The retention of tumor cells in the lung was measured as previously described [70]. Male athymic Balb/c nude mice (5 weeks old) were obtained from Charles River Laboratories Japan. The cells were labeled with 4 µM PKH26 (MINCLARET, Sigma-Aldrich). The animals (7 weeks old) were injected intravenously with 5×10^5^ PKH26-labeled cells. After 24 h, the mice were sacrificed to measure fluorescence intensity of PKH26 extracted from the lungs. The retention of injected cells in the lung was determined by calculating the percentage of the injected fluorescence intensity that was found in the lung extract immediately after injection.

This study was carried out in strict accordance with the recommendations in the Guide for the Care and Use of Laboratory Animals of the National Institutes of Health. The protocol was approved by the Committee on the Ethics of Animal Experiments of the Prefectural University of Hiroshima (Permit Number: H21-002 and H24-017). All surgery was performed under sodium pentobarbital anesthesia, and all efforts were made to minimize suffering.

### Patients and tissue samples

Cancerous tissues and their surrounding normal tissues were obtained from 40 colorectal cancer patients. The samples were initially obtained from surgical resection, endoscopic resection, or biopsy between 2000 and 2004 at Tsuchiya General Hospital in Hiroshima.

The fresh cancerous and normal tissues were lysed and the detergent-soluble cytoplasmic fraction, the detergent-soluble nuclear fraction, and the detergent-insoluble (cytoplasmic- and nuclear-mixed) fraction were generated by subcellular fractionation (Figure S2). Survivin expression was observed in the detergent-insoluble fraction of normal tissues obtained from all patients by immunoblot analysis, but there was no overexpression. On the other hand, although expression levels varied, Survivin overexpression was observed in the detergent-insoluble fraction of cancerous tissues obtained from all patients.

The detergent-soluble cytoplasmic pool of Survivin was quantitated by immunoblot experiments using three different anti-Survivin antibodies (NB 500-201, Novus Biologicals; NB 500-237, Novus Biologicals; sc-10811, Santa Cruz Biotechnology). The Survivin bands in the detergent-soluble cytoplasmic fraction for each antibody were measured by a fluorescence imaging system as described above. The protein levels of Survivin were calculated by the following formula: (estimated detergent-soluble cytoplasmic Survivin level) = (immunofluorescence intensity of Survivin-specific band)/(immunofluorescence intensity of α-tubulin-specific band on the same blot). Three different Survivin antibodies were used for three different blots, and the average level was calculated. Positivity for the detergent-soluble cytoplasmic pool of Survivin was graded according to the protein level of the detergent-soluble cytoplasmic Survivin of colorectal cancer HCT116 cells, as follows: +++ (>60% of HCT116 level); ++ (20–60% of HCT116 level); + (>1% and <20% of HCT116 level); or − (<1% of HCT116 level). The cases showing +++, ++, and + were classified as tumor without the detergent-soluble cytoplasmic Survivin, and the cases showing - were as tumor with the detergent-soluble cytoplasmic Survivin.

This study was reviewed and permitted by both the Tsuchiya General Hospital Human Research Ethics Committee and the Prefectural University of Hiroshima Human Research Ethics Committee. The study protocol followed the ethical guidelines of Tsuchiya General Hospital and Prefectural University of Hiroshima. The individual in this manuscript has given written informed consent (as outlined in the PLoS consent form) to publish these case details.

## Supporting Information

Figure S1 **Outline of the experimental procedure.**
(TIF)

Figure S2 **Schematic illustration of the experimental procedure for detection of Survivin fractionated into the detergent-soluble cytoplasmic fraction in human tissue.**
(TIF)

Table S1 **Tumorigenicity and spontaneous metastatic potential of stable transfectants expressing EGFP and EGFP-Survivin after subcutaneous injection into nude mice_a_.**
(PDF)
