# Supplementary figures and images for: The Detergent-Soluble Cytoplasmic Pool of Survivin Suppresses Anoikis and Its Expression Is Associated with Metastatic Disease of Human Colon Cancer
Source: PLoS One. 2013 Feb 6;8(2):e55710. doi: 10.1371/journal.pone.0055710 (PMC3565976; doi:10.1371/journal.pone.0055710)

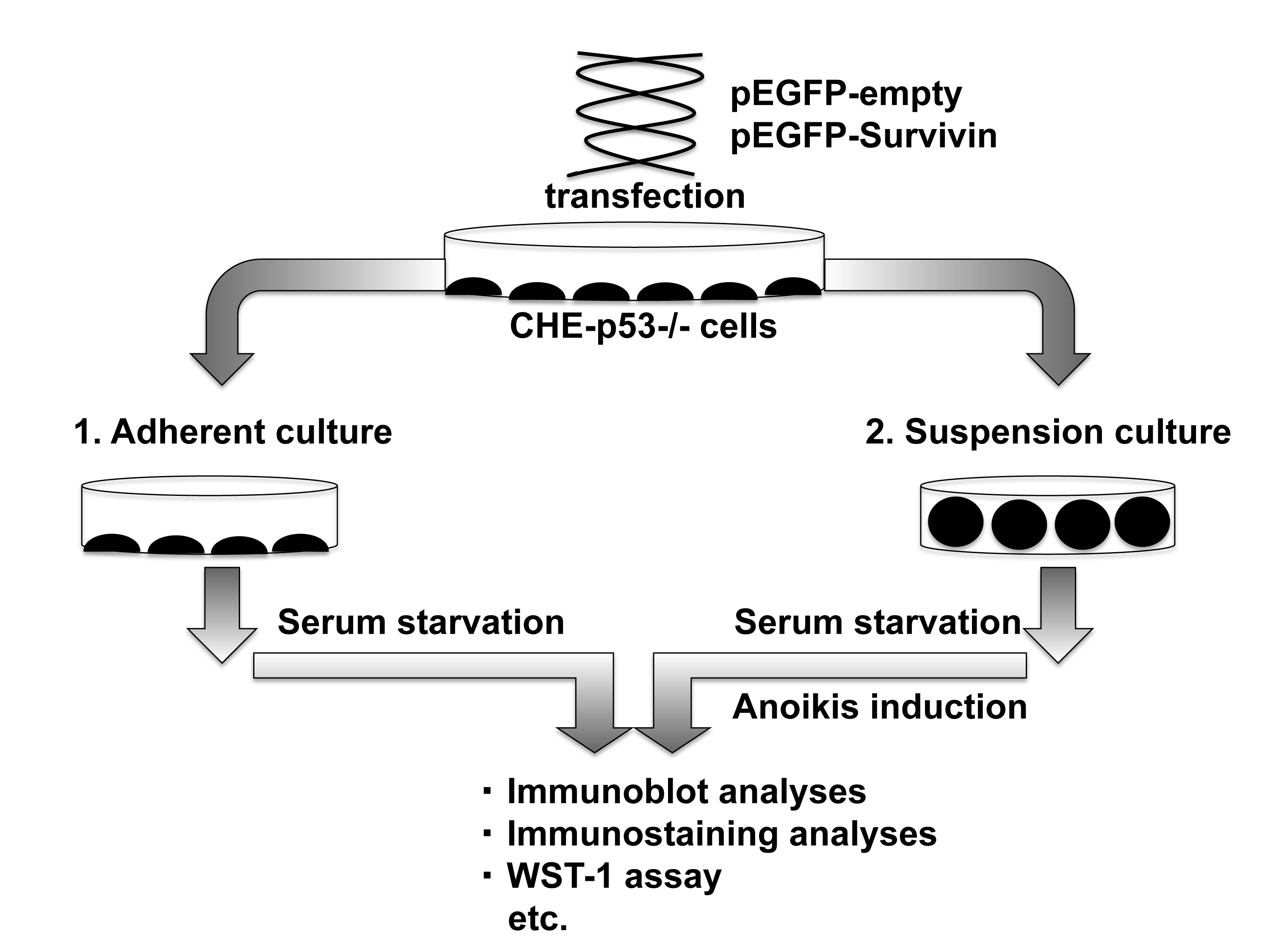

Supplement: Figure S1 — Outline of the experimental procedure. (TIF) [file pone.0055710.s001.tif]

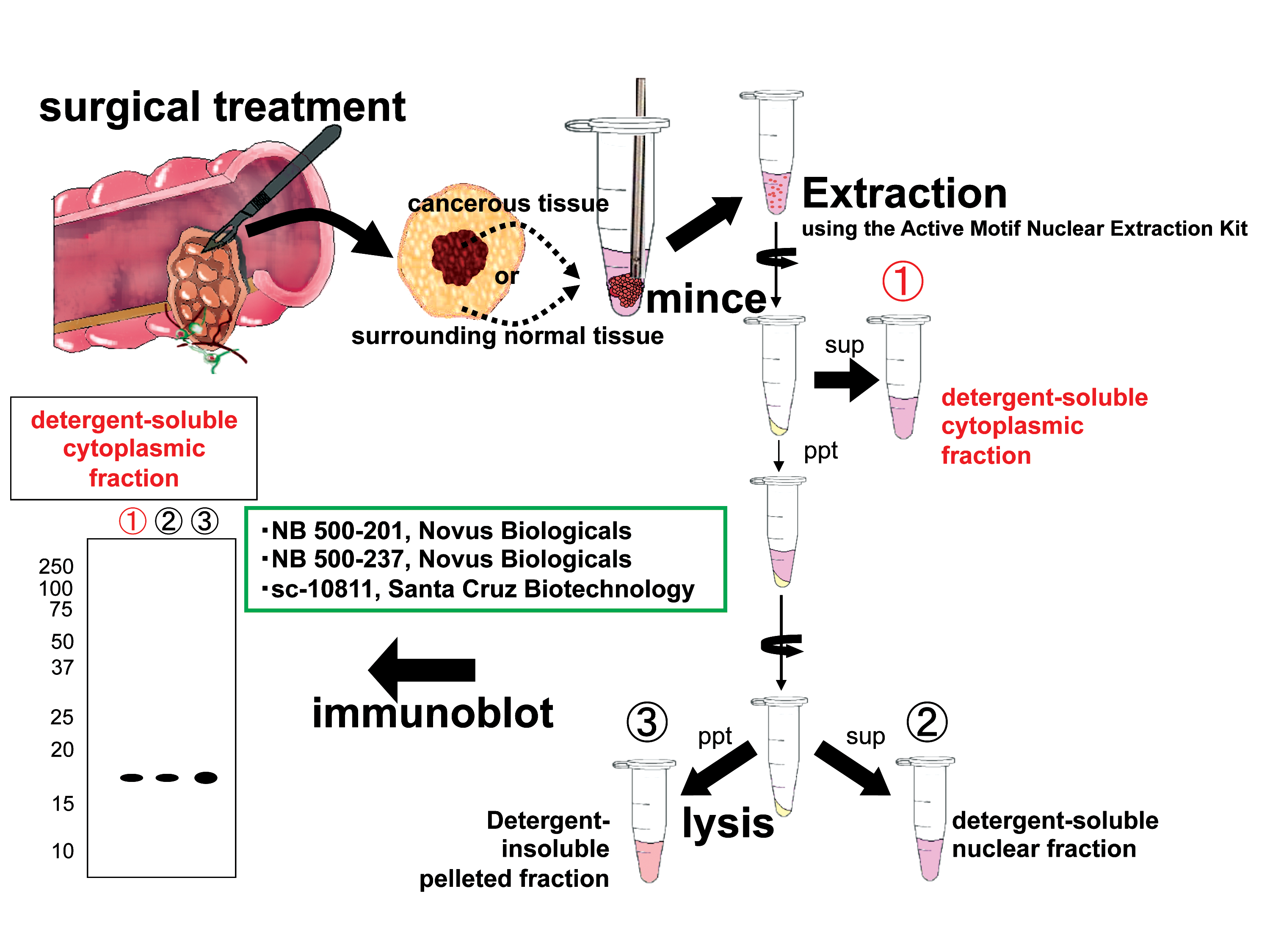

Supplement: Figure S2 — Schematic illustration of the experimental procedure for detection of Survivin fractionated into the detergent-soluble cytoplasmic fraction in human tissue. (TIF) [file pone.0055710.s002.tif]
