# Supplementary material for: The Detergent-Soluble Cytoplasmic Pool of Survivin Suppresses Anoikis and Its Expression Is Associated with Metastatic Disease of Human Colon Cancer
Source: PLoS One. 2013 Feb 6;8(2):e55710. doi: 10.1371/journal.pone.0055710 (PMC3565976; doi:10.1371/journal.pone.0055710)
Supplement: Table S1 — Tumorigenicity and spontaneous metastatic potential of stable transfectants expressing EGFP and EGFP-Survivin after subcutaneous injection into nude micea. (PDF) [file pone.0055710.s003.pdf]

Supplementary Table S1. Tumorigenicity and spontaneous metastatic potential of stable transfectants expressing EGFP and EGFP-Survivin after subcutaneous injection into nude mice[a].

Cell line	Tumorigenicity[b] (No. of tumor-bearing mice/No. of injected mice)	Metastatic potential[c]	
		Lung (No. of mice with metastatic organ/No. of injected mice)	Liver (No. of mice with metastatic organ/No. of injected mice)
EGFP			
Pooled clones	3/8	0/8	0/8
EGFP-Survivin[d]			
Pooled clones	8/8	6/8	5/8

[a]Five-week-old male athymic Balb/c nude mice were obtained from Charles River Laboratories Japan. Experiments were performed in accordance with guidelines of both Committee on Experimental Animals in Prefectural University of Hiroshima and Committee on Experimental Animals in Kanazawa Medical University. [b]EGFP- or EGFP-Survivin-expressing cells (10^7 cells per mouse) were subcutaneously implanted with into the right femoral region of male athymic Balb/c nude mice (6 weeks old). [c]Three months later, the animals were sacrificed to examine all organs for metastases. [d]EGFP-Survivin-expressing cells formed a primary tumor (8/8) and metastasized to the both liver and lungs (6/8 and 5/8).
